# Biological Activities of *Libidibia (Caesalpinia) ferrea* var. *parvifolia* (Mart. ex Tul.) L. P. Queiroz Pod Preparations

**DOI:** 10.1155/2012/514134

**Published:** 2012-05-23

**Authors:** A. C. C. Freitas, N. C. A. Ximenes, J. S. Aguiar, S. C. Nascimento, T. U. L. Lins, L. R. Magalhães, L. C. B. B. Coelho, M. G. Carneiro-da-Cunha, T. Gonçalves-Silva, M. T. S. Correia

**Affiliations:** ^1^Laboratório de Glicoproteínas, Departamento de Bioquímica, Centro de Ciências Biológicas, Universidade Federal de Pernambuco (UFPE), Avenida Professor Moraes Rego s/n, Cidade Universitária, 50670-420 Recife, PE, Brazil; ^2^Laboratório de Bioensaios para Pesquisa de Fármacos, Departamento de Antibióticos, Centro de Ciências Biológicas, Universidade Federal de Pernambuco (UFPE), Avenida Professor Moraes Rego s/n, Cidade Universitária, 50670-420 Recife, PE, Brazil

## Abstract

*Libidibia ferrea* has been used in folk medicine throughout Brazil, and this study evaluated the biological activities of crude extract (CE) as well as a partially purified fraction (F80) obtained from its pods. Results from the MTT assay revealed that only F80 inhibited NCI-H292 cell growth; however, neither CE nor F80 reduced HEp-2 cell growth or sarcoma 180 tumor weight with the *in vivo* assay. Acute oral toxicity of the extract and fraction was evaluated following the steps of Guideline 423, using female mice; LD_50_ for both preparations was determined as 2,500 mg/kg body weight. CE and F80 promoted a reduction of the leukocyte number and nitrite level in inflammatory exudates when the anti-inflammatory assay (carrageenan-induced peritonitis) was performed. CE and F80 inhibited writhing regarding antinociceptive activity (acetic acid-induced writhing response in mice). In conclusion, CE and F80 have no significant cytotoxic or antitumor activities in cell lines showing low toxicity and no action against tumors *in vivo*. Both preparations revealed anti-inflammatory and antinociceptive activities, corroborating the pharmacological basis of *L. ferrea* for ethnomedical use.

## 1. Introduction

Anticancer, pain relief, and anti-inflammatory drugs with weak adverse effects are some of the most important goals in modern research; natural products, especially plant-derivative substances, play a role in this search for an ideal treatment.

*Libidibia ferrea* var. *parvifolia* (Mart. ex Tul.) L. P. Queiroz (Leguminosae), whose basionym is *Caesalpinia ferrea* Mart. ex Tul. (Caesalpiniaceae) [1], is a tree that grows throughout Brazil, especially in the north and northeast regions [2, 3].

*L. ferrea* aqueous and alcoholic preparations are used popularly to treat a number of diseases such as diabetes, rheumatism, and cancer and also are said to litigate diarrhea, inflammation, and pain, among other symptoms [2, 4–7]. Some *L. ferrea* therapeutic properties have been studied including its antitumor effects [6, 8].

The cytotoxic and antitumor activities of an aqueous extract and a fraction obtained from the *L. ferrea *pods as well as preparation effects on the first moments of inflammation and nociception, using *in vitro* and *in vivo* assays, were investigated in light of its ethnomedical applications.

## 2. Materials and Methods

### 2.1. Plant Material

*L. ferrea *pods were collected from Ibimirim City, State of Pernambuco, Northeastern Brazil, in August (period of fruit abundance), 2006, and identified by A. Bocage. A sample of the collected material is archived as voucher specimen number 83566, IPA, at the herbarium “Dárdano de Andrade Lima” (Empresa Pernambucana de Pesquisa Agropecuária, Recife, Brazil).

### 2.2. CE and F80 Preparations

A crude, aqueous extract (CE) was prepared using pod powder in 0.9% NaCl (10% w/v) by gentle shaking for 16 h, at 4°C, passed through gauze, centrifuged (10000 × g) for 15 min, and filtered. Thereafter, proteins were precipitated over 4 h by 0–80% ammonium sulphate fractionation at room temperature; resuspended precipitate was dialyzed against distilled water followed by 0.9% NaCl (F80). Samples were stored at −20°C and subsequently lyophilized. The yield of total dried powder was 25% and 2.94% for CE and F80, respectively.

### 2.3. Animals

Swiss albino female and male mice (*Mus musculus*) weighing approximately 25 g (±50 days old) were obtained from the bioterium of the Departamento de Antibióticos, Universidade Federal de Pernambuco (UFPE) and maintained under constant conditions (temperature: 22 ± 2°C, humidity: 40–60%, 12 h light/12 h dark cycle). The mice were allowed access to standard rodent chow diet (Purina) and water *ad libitum*. These experiments were approved by the Comitê de Ética em Experimentação Animal, Centro de Ciências Biológicas (CEEA-UFPE), Brazil.

### 2.4. Cytotoxic Activity Evaluation

The cell lines used for the *in vitro* cytotoxicity assays were NCI-H292 (human lung mucoepidermoid carcinoma cells) and HEp-2 (human larynx epidermoid carcinoma cells) obtained from the Instituto Adolph Lutz (São Paulo, Brazil). The cells were maintained in Dulbecco's Modified Eagle's medium (DMEM) and supplemented with 10% fetal bovine serum, 2 mM glutamine, 100 U/mL penicillin, and 100 *μ*g/mL streptomycin at 37°C with 5% CO_2_.

**Cell Viability**

Trypan Blue 0.4% (w/v) was used in sodium phosphate buffer (PBS). It penetrates easily into damaged cells staining them blue, while the intact ones remain colorless, allowing determination of living and dead cell percentages. Cells were counted with an inverted LEITZ microscope and a hemocytometer filled with a homogeneous cell suspension aliquot.

**Cytotoxicity**

To determine cytotoxicity, a cell suspension (10^5^ cells/mL) was prepared and distributed in wells of flat-bottom microtiter culture plates. They were incubated in a humidified 5% CO_2_ air atmosphere at 37°C for 24 h; following that, CE and F80 samples at different protein concentrations (50.0; 25.0; 12.5; 6.25 *μ*g/mL) were added and incubated under the same conditions for 72 h [9]. Control wells received only 0.9% sterile NaCl solution; after that, 25 *μ*L of 3-[4,5-dimethylthiazol-2-yl]-2,5-biphenyl tetrazolium bromide (MTT) in PBS (5 mg/mL, w/v) was added, and the plates were maintained at 37°C for 2 h. Culture medium with MTT was suctioned, and dimethylsulphoxide (DMSO) was added to dissolve formazan crystals [10]. MTT assay is based on the capability of living cells to reduce yellow tetrazolium salt into insoluble, purple formazan [11] which precipitates due to the mitochondrial enzyme succinate dehydrogenase active in living cells [12]. Optical density was carried out at 595 nm. Mean optical density (OD) of the test wells was compared to mean OD of control wells to determine IC_50_ (concentration that inhibits 50% of cell growth in relation to control). According to National Cancer Institute (NCI), values of IC_50 _≤ 30 *μ*g/mL, for nonpure products (e.g., extracts), are considered significant [13].

### 2.5. Acute Oral Toxicity

The extract and fraction were evaluated following the steps of Guideline 423 [14] using female mice. Animals were prevented by eating overnight prior to the experiment. Three animals for each preparation were orally given a single dose starting at 300 mg/kg (mg of product/kg of body weight) and observed for 14 days (during the first hour after treatment and then once per day). CE and F80 were dissolved in a 0.9% NaCl sterile saline solution (saline), and the calculation of the exact dose for each mouse was based on individual weights. This first step was repeated in the same way for confirmation of results, and a higher dose (2000 mg/kg) was administrated using different female mice.

### 2.6. Antitumor Activity

Studies were carried out in male mice, six animals per group, aiming to investigate the *in vivo* antitumor activity of CE and F80 against sarcoma 180.

CE, F80, and methotrexate (MTX) were calculated according to animal body weight index (100 mg/kg for CE and F80; 2.5 mg/kg for MTX). Malignant tumor cells (sarcoma 180) from donor animals with 8 days of implanting were used. All animals were previously hygienized in an experimental surgery room. Donor mice were anaesthetized for tumor suctioning, and the ascitic form of the tumor was introduced under the right axilla of the receptor animals. Treatment, by i.p. route, began 24 h after tumor implantation for 7 days. The negative control group received only saline, and the standard group (positive) received MTX as referential antitumor drug. The animals were sacrificed on the eighth day, by cervical dislocation; solid tumors were excised and weighed. Tumor inhibition was expressed as the mean of tumor weight for the treated animal group (T) in comparison to the untreated control group (C). The tumor inhibition was then calculated according to percentage tumor inhibition = [(*C* − *T*)/*C*] × 100. Animal experiments were performed according to the NCI protocol [13].

### 2.7. Anti-Inflammatory Assay—Carrageenan-Induced Peritonitis

Saline (control); the standard drugs: dexamethasone, piroxicam, and indomethacin (2 mg/kg, 3 mg/kg, and 10 mg/kg, resp.); CE; F80 (100 mg/kg for both) were administered by oral route to the correspondent groups (6 animals per group). After one hour, 0.25 mL carrageenan (1% in 0.9% NaCl), intraperitoneally injected, was used as a phlogistic agent. Four hours later, animals were sacrificed by cervical dislocation, and immediately the abdomen was opened [15]. The peritoneal cavity was washed with 2 mL of saline containing 3 mM EDTA. Exudates were collected, and the polymorphonuclear leukocytes (PMNLs) counting was performed in a Neubauer chamber after diluting the sample in Turk solution (0.01% crystal violet in 3% acetic acid).

### 2.8. Nitrite Analysis

Accumulated nitrite (NO_2_^−^) in the peritoneal exudates was measured as an indicator of NO production according to a colorimetric assay based on Griess reaction [16]. The exudates (100 *μ*L) were subjected to reaction with 100 *μ*L Griess reagent (6 mg/mL) at room temperature for 10 min, and then NO_2_^−^ concentration was determined by measuring absorbance at 540 nm. A standard curve was constructed using known concentrations of sodium nitrite (NaNO_2_).

### 2.9. Analgesic Activity—Acetic Acid-Induced Writhing Response

The response to an i.p. injection of acetic acid solution exhibited as a contraction of the abdominal muscles and stretching of hind limbs was evaluated using a method adapted from Young et al. [17]. Animals (6 per group) were pretreated by i.p. with CE or F80 (100 mg/kg), vehicle (saline) or piroxicam (10 mg/kg), and dipyrone (150 mg/kg) as standard drugs. One hour later, a dose of 0.1 mL/10 g body weight of 1% acetic acid was injected via i.p. After 10 min, the number of writhings during the following 20 minute period was counted. Inhibition percentage was calculated through the decrease of total number of writhings in the treated groups against the control group.

### 2.10. Statistical Analysis

Data were expressed as mean ± SEM and statistically assessed using one-way ANOVA (origin 5.0). *P* values less than 0.05 were considered significant.

## 3. Results and Discussion

Natural products, known as secondary metabolites of plants or animals, continue to be an important segment of research into new drugs. In fact, many compounds frequently used in chemotherapy, such as vincristine, taxol, and camptothecins, were isolated from plants or derived from natural prototypes [18–20].

Additionally, the search for new drugs that effectively interfere with the inflammatory process and pain is currently of great relevance; plants traditionally used as well as their derivative substances have historically been valued as a source of anti-inflammatory agents and pain killers [21, 22].

### 3.1. Cytotoxicity

The assays revealed that, at 50.0, 25.0, and 12.5 *μ*g/mL, F80 inhibited NCI-H292 cell growth by 25.6%, 14.3%, and 7.8%, respectively. A concentration of 6.25 *μ*g/mL and HEp-2 cell line induced no growth inhibition. CE did not show significant cytotoxic activity for either cell line. IC_50_ up to 50.0 *μ*g/mL used cell lines did not allow calculation of IC_50_. Therefore, both extract and fraction presented low cytotoxicity.

Only F80 showed some cytotoxic activity; CE did not present cytotoxicity against the cell lines used. Several previous studies have demonstrated that plant extracts and derivative compounds have an anticancer potential *in vitro* or *in vivo.* Nakamura et al. [6] tested distinct *L. ferrea* pod extracts using the *in vitro* Epstein-Barr virus early-antigen activation assay and found that the ethyl acetate extract exhibited the strongest inhibitory activity. Nozaki et al. [23], also working with *L. ferrea*, showed that pauferrol, a compound obtained from the stem, possessed a cell proliferation inhibitory activity through the induction of apoptosis in human leukemia HL60 cells.

### 3.2. Acute Oral Toxicity—LD_50_ Determination

The doses of 300 mg/kg and 2,000 mg/kg did not induce mice weight loss or death from either extract or fraction. LD_50_ cutoff of CE and F80 was determined as 2,500 mg/kg body weight; both concentrations used were considered as safe (category 5) [14].

Despite widespread use (herbal medicine is applied by up to 80% of the population in developing countries), few scientific studies have been undertaken to ascertain the safety and efficacy of traditional remedies [24]. The present investigation suggests that the aqueous extract of *L. ferrea* pods is practically nontoxic via oral route in mice using an acute single dose, indicating that the popular use of this kind of extract can be considered secure.

### 3.3. Antitumor Activity

**Figure 1:**
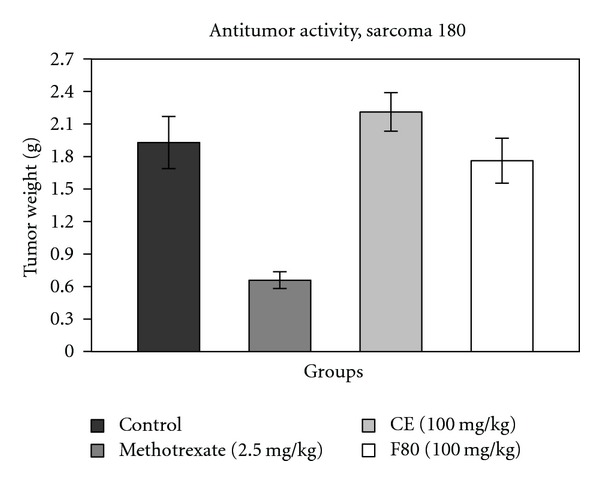
Effects of MTX (methotrexate), CE, and F80 on the growth of sarcoma 180 in Swiss albino male mice. Each column represents the mean of six animals, and vertical lines show the SEM. The asterisk denotes the significance level in comparison to the control value: *P < 0.05.

 F80 showed a tumor growth inhibition of 8.68%, but this value is not significant when compared with the control group. CE did not reveal antitumor activity (Figure 1); CE and F80 did not reduce tumor weight significantly. However, Nakamura et al. [8] isolated two constituents (gallic acid and methyl gallate) from *L. ferrea* pod and tested them against skin carcinogenesis in mice, finding a reduction in the average number of papillomas per mouse. Studying another *Caesalpinia* species, *C. bonducella*, Gupta et al. [18] evaluated the antitumor effect of methanol leaf extract against Ehrlich ascites carcinoma in mice; the extract promoted a significant increase of mean survival time of mice and a decrease of tumor volume, when compared with control group. Then, the antitumor effect of *Caesalpinia* spp. may vary according to extract (and/or isolated constituents) and tumor types used.

### 3.4. Anti-Inflammatory Assay

**Figure 2:**
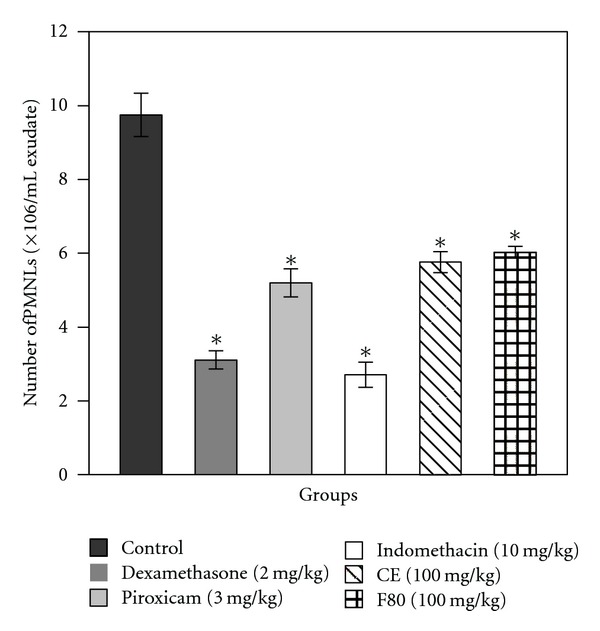
Effect of pretreatment with dexamethasone, piroxicam, indomethacin (standard drugs), CE, and F80 on migration of polymorphonuclear leukocytes (PMNLs) (number of PMNLs/mL exudate) in carrageenan-induced peritonitis in mice. Each column represents the mean of six animals, and vertical lines show the SEM. Asterisks denote the significance level in comparison to the control value: *P < 0.05.

CE and F80 exhibited anti-inflammatory activity, reducing cell migration. CE and F80 showed significant reduction (40.9% and 38.2%, resp.) in the number of PMNL in the inflammatory exudate, similar to piroxicam (46.7%) (Figure 2 and Table 1).

Among the phlogistic agents available (such as dextran, bradykinin, *β*-glucan, etc.), carrageenan is perhaps the most commonly used and well studied [25] producing a maximal edema in 3 h. While the carrageenan model is typically associated with activation of the cyclooxygenase pathway and is sensitive to glucocorticoids and prostaglandin synthesis antagonists, the early phase of the carrageenan response is due to the release of serotonin and histamine [26]. Thus, the significant ameliorative activity of CE and F80 observed in the present study may be due to inhibition of inflammatory mediators such as histamine, serotonin, and prostaglandin and also due to a decrease in NO production.

According to Kelly et al. [27], there is a growing optimism that inhibition of leukocyte recruitment might prevent inappropriate inflammation. So, the search for drugs that act upon cell migration may be of great interest. Our study is unprecedented, since no other study has related the CE and F80 effects on cell migration.

In a previous approach, Carvalho et al. [28] determined the anti-inflammatory activity of a crude aqueous extract of *L. ferrea* pods at 60°C, using the carrageenan-induced paw edema method in mice. Assays revealed that the extract reduced the edema formation significantly from the first moments.

Several plant extracts present expressive anti-inflammatory activity by reducing leukocyte migration. Matos et al. [29], using the same experimental model applied in the present study, also observed a decrease in cell migration in mice treated with an aqueous fraction obtained from a leaf ethanolic extract of *Spiranthera odoratissima*. Gokhale et al. [30] working with ethanolic extracts from *Saussurea lappa*,* Argyreia speciosa, *and* Achyranthes aspera* discovered that all of them show anti-inflammatory activity using the carrageenan-induced paw edema and carrageenan-induced peritonitis models in rats and mice.

### 3.5. Nitrite Analysis

The nitrite content in the exudates was quantified using Griess reagent since nitric oxide (NO) synthesis by inducible nitric oxide synthase (iNOS) is increased in inflammation and leads to cellular injury. This assay is an indirect method to quantify NO, which rapidly reduces to nitrate and nitrite.

Nitric oxide derived from iNOS is involved in various pathological conditions such as inflammation and autoimmune diseases leading to tissue damage [31]. Thus, suppression of iNOS is closely linked with anti-inflammatory action [32].

**Figure 3:**
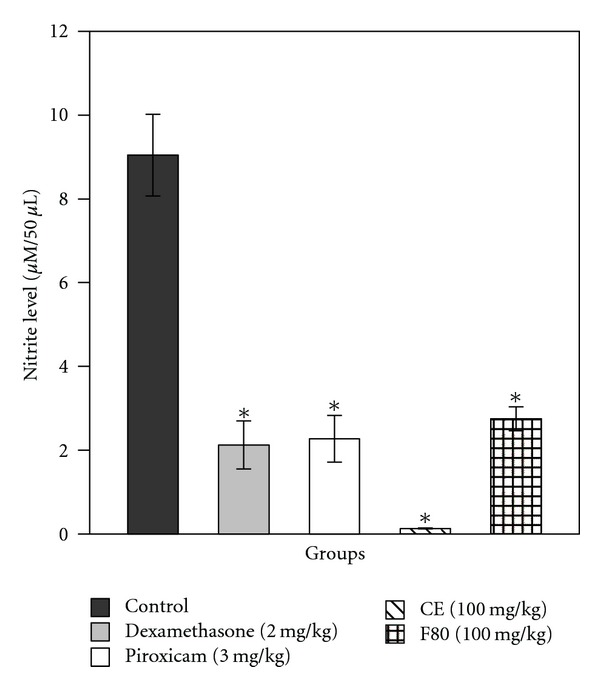
Inhibitory effects of the standard drugs, dexamethasone and piroxicam, CE, and F80 in relation to the control group for NO production. Each column represents the mean of six animals, and vertical lines show the SEM. Asterisks denote the significance level in comparison to the control value: *P < 0.05.

CE and F80 promoted a high reduction in the content of nitrite, especially CE, which decreased it to a level smaller than those presented by the standard drugs, as shown in Figure 3. The strong reduction by CE of nitrite level in the exudate might be attributed to the presence of antioxidant compounds in this aqueous extract. It is likely that CE and F80 show their anti-inflammatory activity through the downregulation of NO production and reduction in leukocyte migration.

Plant extracts showing immunomodulatory activities have been extensively described, especially for their inhibitory effect upon the NO production by macrophages [33, 34]. Ahn et al. [32], investigating the ethanol extract and fractions of *Gastrodia elata* rhizomes, discovered that the extract and its *n*-butanol fraction decreased the nitrite content in the exudates obtained from the carrageenan-induced air-pouch model in mice. Koo et al. [35] also verified this reduction by testing two constituents (geniposide and genipin) obtained from the ethanol extract of *Gardenia jasminoides* fruit in rats.

### 3.6. Analgesic Activity—Acetic Acid-Induced Writhing Response

 There are different routes to evaluate nociception; however, the assay using intraperitoneally acetic acid was the chosen approach. The animals were pretreated one hour before administration of phlogistic agent, then apparently, there was no influence of the administration effect. Other authors have also used parenteral route in their researches [36, 37].

 It is well known that the intraperitoneal administration of agents that irritate serous membranes, such as acetic acid, causes a stereotypical behavior in mice characterized by abdominal contractions, movements of the body as a whole, twisting of dorsoabdominal muscles, and a decrease in motor activity and coordination [38]. Using the acetic acid-induced writhing response, which is the visceral pain model, the analgesic mechanism of abdominal writhing involves different nociceptive mechanisms, such as the process or release of arachidonic acid metabolites via cyclooxygenase and prostaglandin biosynthesis, opioid mechanisms, local peritoneal receptors and mediators related to acetylcholine and histamine, and sympathetic system mediators [35, 39, 40].

Acetic acid causes algesia by releasing endogenous substances, which then excite the pain nerve endings; the abdominal constriction is related to sensitization of nociceptive receptors to prostaglandins [41]. Although this assay is nonspecific (e.g., anticholinergic, antihistaminic, and other agents also show activity), it is a very sensitive procedure that enables the detection of peripheral antinociceptive activity of compounds using animal protocols and is widely used for analgesic screening. This method is simple and reliable and affords rapid evaluation of peripheral analgesic action [42].

Dipyrone (metamizol) is a widely used nonsteroidal anti-inflammatory drug (NSAID). In addition to the well-known peripheral effects of NSAIDs—especially prostaglandin synthesis inhibition—and the fact that dipyrone is able to induce a significant antinociceptive effect in the absence of an anti-inflammatory response, it has been proposed that it produces antinociception at least partially by acting upon central nervous system structures [43, 44].

**Figure 4:**
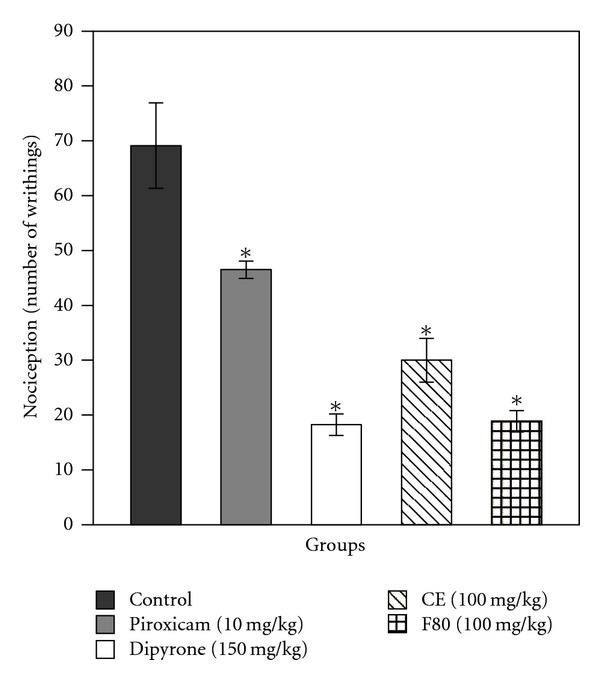
Effects of the standard drugs, piroxicam and dipyrone, CE, and F80 in relation to the control group on writhing induced in mice by intraperitoneal injection of acetic acid. Each column represents the mean of six animals, and vertical lines show the SEM. Asterisks denote the significance level in comparison to the control value: *P < 0.05.

Since CE and F80 were confirmed to have anti-inflammatory activity in carrageenan-induced peritonitis (Figure 2 and Table 1), their analgesic activity was examined. As shown in Table 2 and Figure 4, both preparations protected mice against chemically induced noxious stimulus, causing an inhibition at the rate of 56.6% (for CE, higher than that promoted by piroxicam) and 72.7% (for F80, comparable to dipyrone) on the writhing response induced by acetic acid. These results indicate that CE and F80 showed analgesic action in addition to anti-inflammatory activity. Subsequently, it suggests that prostaglandin biosynthesis might be commonly involved in both activities of CE and F80 or that the mode of action of both preparations is related to sensitization of nociceptive receptors to prostaglandins.

Carvalho et al. [28] also evaluated the analgesic activity of an L. ferrea pod extract, using a variation of the method described here and the hot-plate test in mice. Our results corroborate theirs, both showing significant reduction in nociception in treated groups.

## 4. Conclusions

CE and F80 have neither significant cytotoxic nor antitumor activities over NCI-H292 and HEp-2 cell lines or the solid tumor (sarcoma 180) assayed. However, further studies are necessary to evaluate these activities using other cell lines and tumors to elucidate whether these preparations are effective against any of them, as this plant is used popularly for cancer prevention. On the other hand, their low toxicity allows the use of CE and F80 with a degree of safety in other situations. Furthermore, the antinociceptive and anti-inflammatory effects demonstrated in the present study contributed to the ethnomedical uses of *L. ferrea*. Further investigations are necessary to elucidate the precise mechanisms of action and the compounds responsible for these effects.

## Figures and Tables

**Table 1:** Evaluation of anti-inflammatory activity of standard drugs (dexamethasone, piroxicam and indomethacin), CE, and F80 on carrageenan-induced peritonitis in pretreated mice.

Compound	PMNL/mL exudate ± SEM (×10^6^)	Anti-inflammatory activity (%)
Control	9.7 ± 0.8	—
Dexamethasone (2 mg/kg)	3.1 ± 0.3*	68.1
Piroxicam (3 mg/kg)	5.2 ± 0.5*	46.7
Indomethacin (10 mg/kg)	2.7 ± 0.4*	72.2
CE (100 mg/kg)	5.8 ± 0.3*	40.9
F80 (100 mg/kg)	6.0 ± 0.1*	38.2

*n* = 6. *P < 0.05 versus the control group.

**Table 2:** Antinociceptive effect of standard drugs (piroxicam and dipyrone), CE, and F80 on acetic acid-induced writhing response test in mice.

Compound	Dose	Medium ± SEM	Protection (%)
Control	—	69.1 ± 7.8	—
Piroxicam	10 mg/kg	46.5 ± 1.6*	32.73
Dipyrone	150 mg/kg	18.2 ± 1.9*	73.60
CE	100 mg/kg	30.0 ± 4.0*	56.60
F80	100 mg/kg	18.9 ± 1.9*	72.72

*n* = 6. *P < 0.05 versus the control group.
